# PHD2 regulates arteriogenic macrophages through TIE2 signalling

**DOI:** 10.1002/emmm.201302695

**Published:** 2013-04-25

**Authors:** Alexander Hamm, Lorenzo Veschini, Yukiji Takeda, Sandra Costa, Estelle Delamarre, Mario Leonardo Squadrito, Anne-Theres Henze, Mathias Wenes, Jens Serneels, Ferdinando Pucci, Carmen Roncal, Andrey Anisimov, Kari Alitalo, Michele De Palma, Massimiliano Mazzone

**Affiliations:** 1 Laboratory of Molecular Oncology and Angiogenesis, Vesalius Research CenterVIB, Leuven, Belgium; 2 Laboratory of Molecular Oncology and Angiogenesis, Vesalius Research Center, Department of OncologyKU Leuven, Leuven, Belgium; 3 Laboratory of Angiogenesis and Neurovascular Link, Vesalius Research CenterVIB, Leuven, Belgium; 4 Laboratory of Angiogenesis and Neurovascular Link, Vesalius Research CenterKU Leuven, Leuven, Belgium; 5 Life and Health Sciences Research Institute, Minho UniversityBraga, Portugal; 6 The Swiss Institute for Experimental Cancer Research (ISREC), School of Life Sciences, Swiss Federal Institute of Technology Lausanne (EPFL)Lausanne, Switzerland; 7 Angiogenesis and Tumor Targeting Unit, and HSR-TIGET, San Raffaele Scientific InstituteMilan, Italy; 8 Atherosclerosis Research Laboratory, CIMA-University of NavarraPamplona, Spain; 9 Wihuri Research Institute and Translational Cancer Biology Program, Biomedicum Helsinki, University of HelsinkiHelsinki, Finland

**Keywords:** arteriogenesis, ischaemia, macrophages, PHD2, TIE2

## Abstract

Occlusion of the main arterial route redirects blood flow to the collateral circulation. We previously reported that macrophages genetically modified to express low levels of prolyl hydroxylase domain protein 2 (PHD2) display an arteriogenic phenotype, which promotes the formation of collateral vessels and protects the skeletal muscle from ischaemic necrosis. However, the molecular mechanisms underlying this process are unknown. Here, we demonstrate that femoral artery occlusion induces a switch in macrophage phenotype through angiopoietin-1 (ANG1)-mediated *Phd2* repression. ANG blockade by a soluble trap prevented the downregulation of *Phd2* expression in macrophages and their phenotypic switch, thus inhibiting collateral growth. ANG1-dependent *Phd2* repression initiated a feed-forward loop mediated by the induction of the ANG receptor TIE2 in macrophages. Gene silencing and cell depletion strategies demonstrate that TIE2 induction in macrophages is required to promote their proarteriogenic functions, enabling collateral vessel formation following arterial obstruction. These results indicate an indispensable role for TIE2 in sustaining *in situ* programming of macrophages to a proarteriogenic, M2-like phenotype, suggesting possible new venues for the treatment of ischaemic disorders.

→See accompanying articles http://dx.doi.org/10.1002/emmm.201302752 and http://dx.doi.org/10.1002/emmm.201302794

## INTRODUCTION

Vascular stenosis reduces blood supply resulting in ischaemia, which causes tissue demise and dysfunction. This condition is, however, associated with the formation of new blood vessels (angiogenesis) and remodelling of preexisting collateral arterioles (arteriogenesis), which reestablish blood flow to the downstream tissue (Schirmer et al, 2009; Simons & Ware, 2003). Spontaneous angiogenesis and arteriogenesis thus attenuate local tissue ischaemia and improve the clinical outcome of the disease (Schirmer et al, 2009; Schultz et al, 1999; Yu et al, 2005). Upon occlusion of an artery, the blood flow is redirected into preexisting arteriolar anastomoses, causing enhanced shear stress on the endothelium of the collateral circulation (Heil & Schaper, 2004; Schaper, 2009; van Royen et al, 2003). As a consequence, endothelial cells (ECs) secrete vascular endothelial growth factor (VEGF), which induces the production of chemokine (C-C) motif ligand-2 (CCL2, or MCP1) from the endothelium or adjacent smooth muscle cells (SMCs), leading to monocyte recruitment (Heil et al, 2002; Ito et al, 1997b; Schirmer et al, 2009). Once in the periarteriolar region, macrophages produce growth factors that enhance the motility and proliferation of SMCs, as well as proteases that digest the extracellular matrix to provide space for newly recruited SMCs (Heil & Schaper, 2004; Schirmer et al, 2009).

Cells belonging to the macrophage lineage have long been recognized to be heterogeneous (Sica & Mantovani, 2012). Because lineage-defined subsets have not been unambiguously identified to date, macrophage heterogeneity is likely to reflect their plasticity in response to microenvironmental signals. In cancer, atherosclerosis, or developing tissues, macrophages may display an ‘alternatively activated’ (or M2-like) phenotype, which enhances debris scavenging, angiogenesis and tissue remodelling (Sica & Mantovani, 2012). Among the markers that characterize this macrophage phenotype, the angiopoietin (ANG) receptor TIE2 has received particular attention because it mediates the association of M2-like macrophages with blood vessels and regulates their ability to induce angiogenesis in both physiological and pathological conditions (Capobianco et al, 2011; De Palma et al, 2005; Fantin et al, 2010; Mazzieri et al, 2011; Pucci et al, 2009).

By using genetic tools in mice, we have recently shown that heterozygous deletion of *Egln1*, which encodes for the oxygen-sensitive prolyl hydroxylase domain (PHD) protein 2 (PHD2), promotes a proarteriogenic, M2-like phenotype in macrophages, characterized by enhanced expression of the macrophage mannose receptor (MRC1/CD206) and TIE2. This switch in the macrophage phenotype relied on activation of the canonical NF-κB pathway and promoted the maturation of collateral arterial branches, allowing blood flow to downstream tissues in case of occlusion of the main arterial route and thus providing protection against ischaemic damage (Takeda et al, 2011). In macrophages, PHD-containing enzymes (*i.e.* PHD1, PHD2 and PHD3) utilize oxygen to negatively regulate the hypoxia-inducible factors HIF-1 and HIF-2, as well as NF-κB-mediated signals (Escribese et al, 2012; Kiss et al, 2012; Takeda et al, 2009; Takeda et al, 2011). Since initiation of arteriogenesis by macrophages takes place in a non-hypoxic environment distant from the ischaemic area (Gray et al, 2007; Ito et al, 1997a), oxygen will not be limiting for the function of PHDs in this condition. This raises important questions regarding the physiological relevance of decreased PHD2 activity in normoxia; its role in promoting the proarteriogenic, M2-like phenotype of macrophages; and the identity of the molecular players that trigger this specific macrophage differentiation state during arteriogenesis.

In the current study, we characterize the mechanisms governing oxygen-independent regulation of PHD2 in macrophages following femoral artery occlusion, and the molecular pathways that orchestrate their proarteriogenic phenotype.

## RESULTS

### *Phd2* downregulation by ANG1 promotes proarteriogenic macrophages

**Figure 1:**
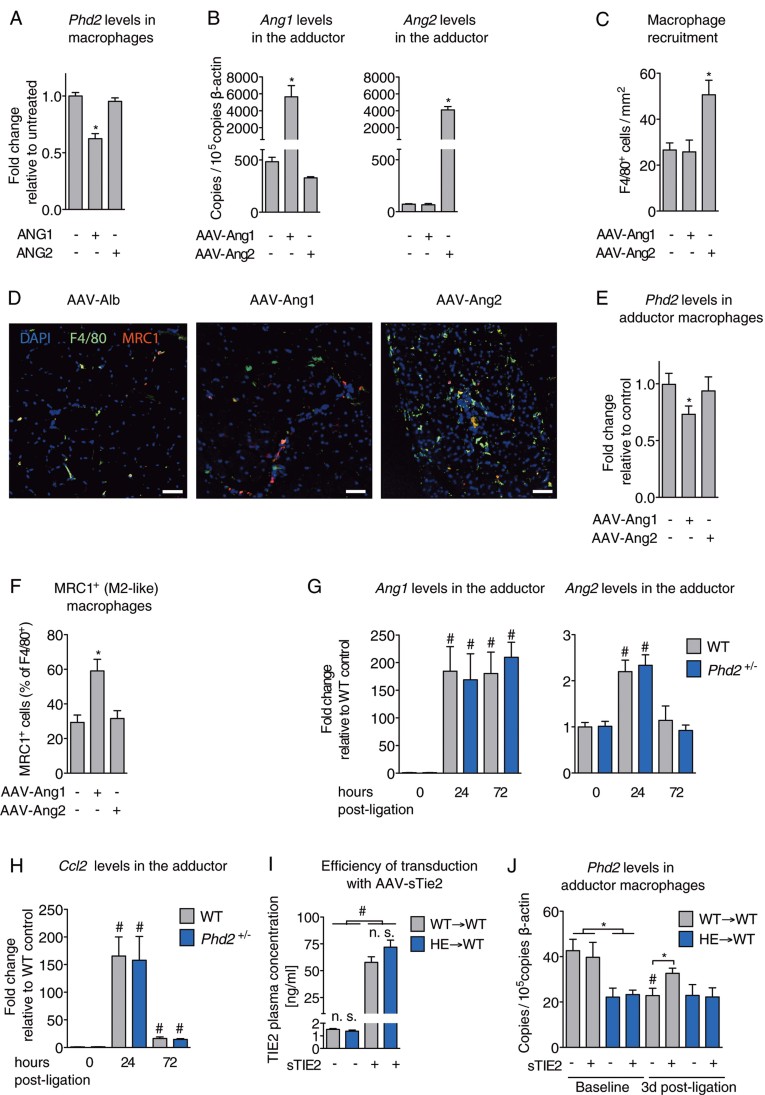
*Phd2* downregulation is dependent on ANG1 *p < 0.05 towards all other groups in A–D, F, towards indicated bars in I,J; ^#^*p* < 0.05 towards baseline in G,H,J., towards control in I; n.s., not significant (*p* > 0.05). *Phd2* mRNA levels in peritoneal macrophages upon stimulation with Angiopoietin-1 (ANG1) or Angiopoietin-2 (ANG2) (*n* = 4).*Ang1* and *Ang2* mRNA levels in adductor muscle 14 days after administration of an AAV encoding for *Ang1* (AAV-Ang1), *Ang2* (AAV-Ang2) or Albumin (AAV-Alb, [−]) as control (*n* = 5).Overall recruitment of F4/80^+^ macrophages upon overexpression of *Ang1* (AAV-Ang1) or *Ang2* (AAV-Ang2) (*n* = 5).Immunofluorescence staining for F4/80 and MRC1 on adductor sections (scale bars, 50 µm).*Phd2* mRNA levels in adductor macrophages upon overexpression of *Ang1* (AAV-Ang1) or *Ang2* (AAV-Ang2) (*n* = 5).Overexpression of *Ang1* (AAV-Ang1), but not *Ang2* in the adductor muscle increases the relative proportion of MRC1^+^ (M2-like) macrophages (*n* = 5).*Ang1* and *Ang2* mRNA levels in WT and *Phd2*^+/−^ adductor muscle at baseline (0 h), 24 h, and 72 h after femoral artery ligation (*N* = 4–8).*Ccl2* mRNA levels in WT and *Phd2*^+/−^ adductor muscle at baseline (0 h), 24 h, and 72 h after femoral artery ligation (*N* = 4–8).Plasma concentrations of sTIE2 in WT mice transplanted with BM cells from WT (WT → WT) or *Phd2*^*+/−*^ (HE → WT) mice as readout of efficiency of transduction by AAV encoding for a soluble angiopoietin trap (sTIE2; indicated as [+]) or albumin as control (indicated as [−]) (*N* = 6–8).ANG blockade prevents *Phd2* downmodulation in WT macrophages in ischaemia. *Phd2* mRNA levels in adductor macrophages from BM-transplanted (BMT) mice (WT → WT and HE → WT, *N* = 4–5).

By using a mouse model of hindlimb ischaemia, we previously showed that wild-type (WT) macrophages isolated from the adductor muscle, where collateral arteriogenesis occurs upon femoral artery occlusion, display reduced expression of *Phd2*, thus resembling *Phd2*^+/−^ macrophages in treatment-naïve mice (Takeda et al, 2011). Consistent with previous studies (Gray et al, 2007; Ito et al, 1997a), femoral artery occlusion did not affect the availability of oxygen (one of the substrates required for the activity of PHDs) in the proximity to the ligation point, while it did so in the lower limb (Supporting Information Fig S1A). Therefore, the amount of PHD2, and not oxygen availability, should limit its activity in the proximity to the ligation point. Since ANG signalling has been previously reported to reduce PHD2 protein levels and activity (Chen & Stinnett, 2008), we hypothesized that acute ANG release following femoral artery occlusion could be responsible for the reduction of *Phd2* transcripts in WT macrophages, hence favouring their switch to a proarteriogenic phenotype (Takeda et al, 2011). *In vitro* stimulation with ANG1 but not ANG2 resulted in about 40% decrease of *Phd2* transcripts in WT macrophages (Fig 1A). We then used adeno-associated vectors (AAVs) to overexpress either ANG1 or ANG2 in the adductors of WT mice. Both transgenes were strongly expressed at comparable levels (Fig 1B). In this setting, ANG1 did not affect muscle infiltration by F4/80^+^ macrophages (Fig 1C and D), whereas ANG2 greatly increased their numbers (Fig 1C and D), possibly through indirect effects mediated by changes in the microvasculature (Fiedler et al, 2006; Roviezzo et al, 2005). Consistent with our *in vitro* data, ANG1 but not ANG2 significantly reduced the levels of *Phd2* by about 30% in macrophages sorted from the adductors of these mice (Fig 1E), and increased their expression of the M2-like marker, MRC1, compared to mock controls (Fig 1D and F).

We then quantified the expression of *Ang1* and *Ang2* mRNAs in the adductor during the time when macrophages are recruited to the pericollateral area (*i.e.* 24–72 h post-ligation) upon induction of ischaemia. The two cytokines were similarly expressed at baseline in both WT and *Phd2*^+/−^ mice (Fig 1G). Twenty-four hours after ligation, *Ang1* and *Ang2* were comparably upregulated (vs. baseline) in the two genotypes. While *Ang1* was strongly induced and remained high 72 h post-ligation, *Ang2* returned to baseline levels in both WT and *Phd2*^+/−^ mice (Fig 1G). *Ccl2*, one of the most important macrophage chemoattractants during limb ischaemia (Fujii et al, 2006; Heil & Schaper, 2004; Ito et al, 1997b; Nickerson et al, 2009), was not differentially expressed in both genotypes at baseline and was comparably induced after femoral artery occlusion (Fig 1H).

Finally, we assessed the *in vivo* relevance of ANG-mediated *Phd2* regulation in macrophages. To this end, WT recipient mice were reconstituted with bone marrow (BM) cells from WT or *Phd2*^+/−^ mice (WT → WT and HE → WT, respectively), and then treated with AAVs that express either an ANG-trap consisting of the extracellular domain of TIE2 (AAV-sTIE2) or albumin (AAV-Alb) as control. The resulting plasma levels of sTIE2 were more than 40 times higher in AAV-sTIE2 than AAV-Alb injected mice; no significant differences were observed between genotypes (Fig 1I). Ten days after AAV administration, we sorted F4/80^+^ tissue-resident macrophages from adductors, both at baseline and 72 h post-ligation, and measured the transcript levels of *Phd2*. As previously observed (Takeda et al, 2011), the levels of *Phd2* were halved in WT macrophages after ischaemia, thus resembling the levels of *Phd2* in *Phd2*^+/−^ macrophages at baseline (Fig 1J). Strikingly, ANG blockade prevented this effect in WT macrophages, whereas it was ineffective in *Phd2*^+/−^ macrophages (Fig 1J). At baseline, *Phd2* levels were not changed by sTIE2 in either WT or *Phd2*^+/−^ macrophages.

**Figure 2:**
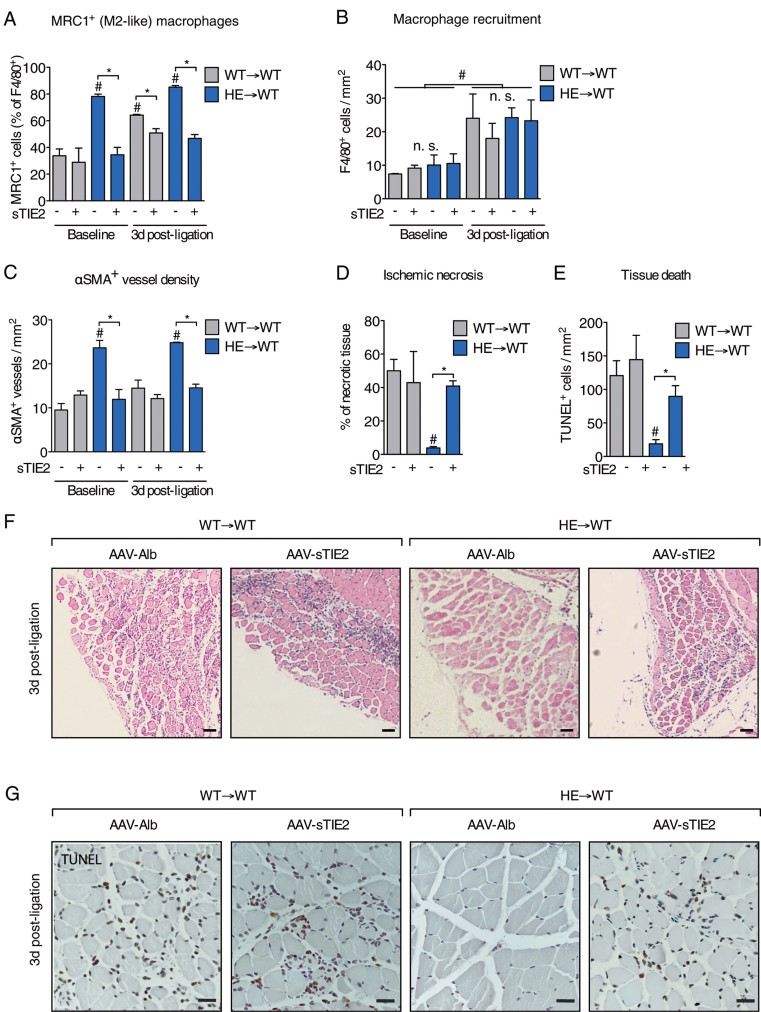
ANG signalling triggers a proarteriogenic (M2-like) macrophage phenotype without affecting macrophage recruitment ANG blockade abrogates protection against ischaemic necrosis by abolishing collaterogenesis. Analyses are performed upon administration of an AAV encoding for a soluble angiopoietin trap (AAV-sTIE2; indicated as [+]) or albumin as control (AAV-Alb; indicated as [−]) in WT mice transplanted with BM cells from WT (WT → WT) or *Phd2*^+/−^ (HE → WT) mice, both at baseline and 3 days after ligation. *p < 0.05; ^#^*p* < 0.05 towards WT control at baseline in A,B, towards baseline in C, towards WT control in D,E. sTIE2 abrogates the proarteriogenic macrophage phenotype, as shown by microscopic quantification of MRC1^+^, M2-like macrophages (% of F4/80^+^ macrophages) in adductors (*N* = 4–5).Quantification of F4/80^+^ macrophages on immunostained sections of adductors. n.s.: not significant (*p* > 0.05).Quantification of αSMA^+^ vessels on immunostained sections of adductors (*N* = 4–5).Quantification of ischaemic necrosis (H&E) in crural muscles (*N* = 4–5).Quantification of ischaemic necrosis (TUNEL) in crural muscles (*N* = 4–5).H&E staining of crural muscles (scale bars, 100 µm).Representative micrographs of TUNEL-stained crural muscles (scale bars, 50 µm).

We then assessed how sTIE2 affects the recruitment and phenotype of adductor macrophages at 72 h post-ligation, when direct effects of ANGs on ECs and angiogenesis should not be relevant. Under these conditions, sTIE2-mediated ANG blockade prevented macrophage skewing towards the M2-like (MRC1^+^) phenotype in ligated WT → WT mice and both ligated and non-ligated HE → WT mice (Fig 2A), whereas it did not affect the total number of infiltrating F4/80^+^ macrophages in either genotype (Fig 2B). Following occlusion of the main arterial route, preexisting collateral branches undergo an extensive remodelling consisting of the massive recruitment and proliferation of alpha-smooth muscle actin (αSMA)-positive SMCs (Schaper, 2009). sTIE2 prevented the maturation of αSMA-positive blood vessels in HE → WT mice both at baseline and 72 h post-ligation (Fig 2C and Supporting Information Fig S2A), Likewise, the protection from ischaemic necrosis and tissue death in the crural muscle of HE → WT mice was abrogated by sTIE2 treatment (Fig 2D–G and Supporting Information Fig S2B).

Together, these results suggest that following obstruction of the main artery, ANG1-mediated TIE2 signalling induces macrophages to acquire a proarteriogenic phenotype, which promotes collateral vessel maturation. This phenotype was phenocopied at baseline by genetic deletion of one *Phd2* allele in macrophages.

### TIE2 is required to sustain the proarteriogenic phenotype of macrophages

**Figure 3:**
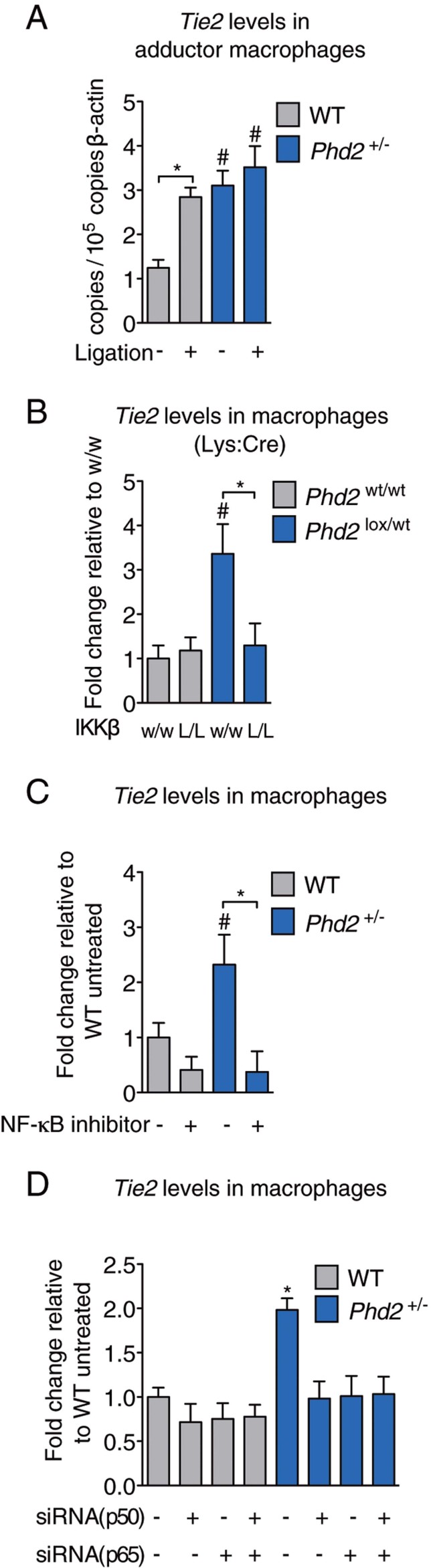
*Tie2* is upregulated in *Phd2*^+/−^ macrophages via NF-κB *p < 0.05; ^#^*p* < 0.05 towards WT baseline in A, towards *Phd2*^wt/wt^ in B, towards WT control in C. **A.** *Tie2* mRNA levels in WT and *Phd2*^+/−^ adductor macrophages at baseline (−) and 72 h (+) after ligation (*N* = 4–6).**B,C.** *Tie2* mRNA levels in WT (*Phd2*^wt/wt^) and *Phd2*^+/−^ (*Phd2*^lox/wt^) peritoneal macrophages after genetic inactivation (L/L for inactivation vs. w/w for wild-type) of IKKκ (B), or pharmacological inhibition of NF-κB (C) (*N* = 4).**D.** Silencing of the canonical subunits p65 (*Rela*) or p50 (*Nfkb1*) inhibits NF-κB hyperactivation in *Phd2*^*+/−*^ macrophages and combined knockdown of both subunits restores NF-κB function back to the WT levels (*N* = 4).

Previous studies showed that TIE2 is expressed at low level in circulating monocytes but is upregulated upon their differentiation into perivascular, M2-like macrophages (De Palma et al, 2008; Pucci et al, 2009). In F4/80^+^ macrophages freshly isolated from adductor muscles, transcript levels of *Tie2* (*i.e. Tek*) at baseline were 2.5 times higher in *Phd2*^+/−^ versus WT mice (Fig 3A). After ligation, *Tie2* transcript levels were augmented only in WT macrophages, likely because *Phd2*^+/−^ macrophages display increased *Tie2* expression already at baseline (Fig 3A). At the molecular level, *Tie2* induction by reduced *Phd2* levels was dependent on the activation of the canonical NF-κB pathway. Indeed, genetic disruption or pharmacological inhibition of NF-κB prevented the upregulation of *Tie2* in *Phd2*^+/−^ macrophages (Fig 3B and C). A similar effect was obtained upon silencing of each of the two subunits belonging to the canonical NF-κB pathway p65 (*i.e. Rela*) and p50 (*i.e. Nfkb1*) (Fig 3D) to approximately 50% (Supporting Information Fig S3A), suggesting the importance of the p65/p50 heterodimer in the induction of *Tie2* expression in *Phd2*^+/−^ macrophages.

We then asked whether the upregulation of *Tie2* in macrophages was required to sustain the proarteriogenic phenotype of *Phd2*^+/−^ macrophages at baseline and WT macrophages in ischaemia. To address this question, we silenced *Tie2* specifically in WT and *Phd2*^+/−^ mature haematopoietic cells by using a previously described genetic strategy (Mazzieri et al, 2011). We employed lentiviral vectors (LVs) that express an artificial microRNA (amiR) either against *Tie2* (amiR(*Tie2*)) or *Luciferase* (amiR(*Luc*)) from a tetracycline-responsive element (TRE)-containing promoter, which also controls the expression of a marker gene (orange fluorescent protein, OFP). To study the effect of *Tie2* knockdown in macrophages *in vivo*, we transduced WT or *Phd2*^+/−^ BM-derived haematopoietic stem/progenitor cells (HSPCs) with either amiR(*Tie2*) or amiR(*Luc*) LVs, followed by transduction with a second LV carrying the reverse tetracycline transactivator (rtTA-m2) under the control of the phosphoglycerate kinase (PGK) promoter. In order to prevent rtTA expression and *Tie2* knockdown in HSPCs, which require TIE2 for their survival and clonogenic activity (Arai et al, 2004), the rtTA expression cassette was modified by incorporating target sequences for the HSPC-specific miR-126 (Gentner et al, 2010) in the 3′-UTR (to obtain the PGK:rtTA-m2:miR-126T LV), as described previously (Mazzieri et al, 2011). Double transduced HSPCs were then used to reconstitute the haematopoietic system of lethally irradiated WT recipient mice (WT amir(*Tie2*)-BMT or *Phd2*^+/−^ amir(*Tie2*)-BMT; and WT amir(*Luc*)-BMT or *Phd2*^+/−^ amir(*Luc*)-BMT). Upon doxycycline administration, haematopoietic reconstitution with OFP^+^ haematopoietic cells, which express the amiR, was about 40% in all the groups (Supporting Information Fig S4A and B). Silencing of *Tie2* expression in the OFP^+^ fraction was about 75% in both WT amir(*Tie2*)-BMT and *Phd2*^+/−^ amir(*Tie2*)-BMT when compared to WT amir(*Luc*)-BMT and *Phd2*^+/−^ amir(*Luc*)-BMT mice (Supporting Information Fig S4C).

**Figure 4:**
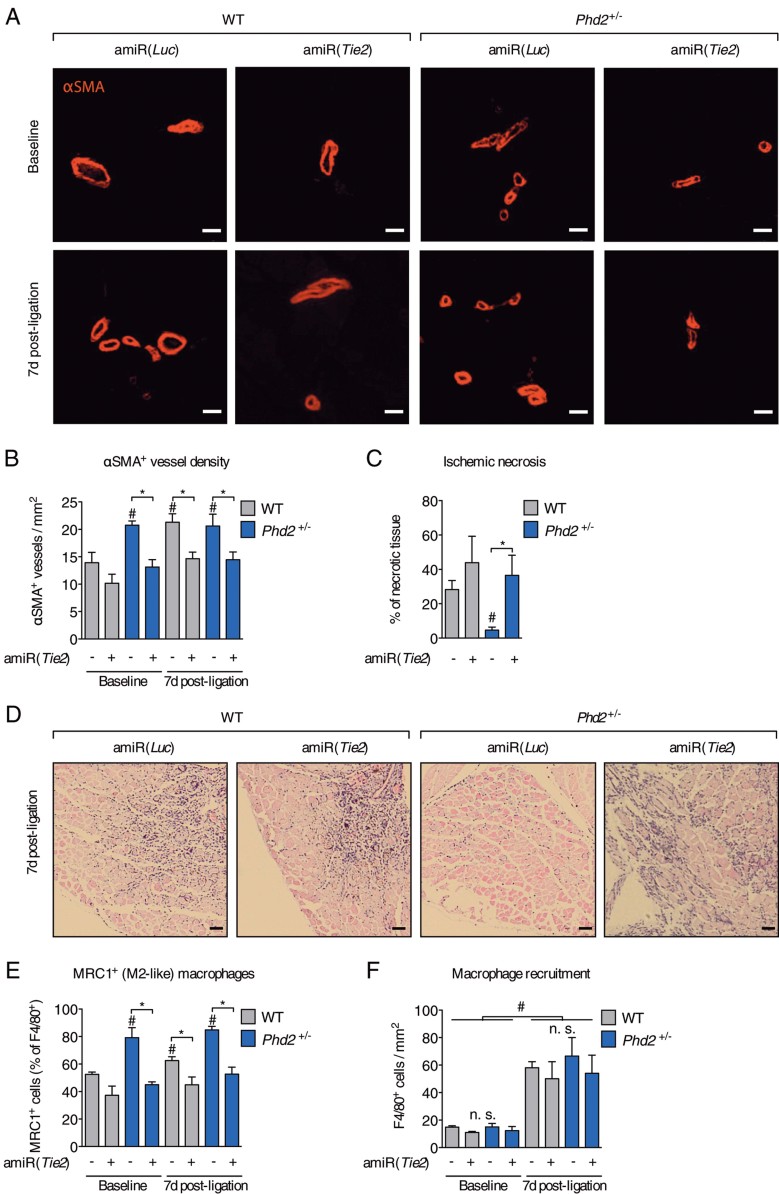
TIE2 is required for proarteriogenic macrophage skewing All analyses are performed at baseline or 7 days after femoral artery ligation, 6 weeks after BMT of WT mice with HSPCs from WT or *Phd2*^+/−^ mice expressing an artificial miR against *Tie2* (amiR(*Tie2*); +) or a control amiR (amiR(*Luc*); −), specifically in mature haematopoietic cells (*N* = 4–5). *p < 0.05; ^#^*p* < 0.05 towards WT control baseline in B,E, towards WT control in C, towards baseline group in F; n.s.: not significant (*p* > 0.05). Immunofluorescence staining of αSMA^+^ vessels in adductors (scale bars, 20 µm).Quantification of αSMA^+^ vessels in adductors: *Tie2* silencing abrogates ischaemia-induced αSMA^+^ vessel formation in WT BMT mice and increases αSMA^+^ SMC vessel coverage at baseline in *Phd2*^+/−^ BMT mice (*N* = 4–5).Quantification of ischaemic necrosis in crural muscles (*N* = 4–5).H&E staining of crural muscles (scale bars, 100 µm).*Tie2* silencing abrogates macrophage phenotype switch towards an M2-like phenotype in WT BMT mice after ligation and *Phd2*^+/−^ BMT mice at baseline, as assessed by the relative proportion of MRC1^+^ macrophages in the adductors.*Tie2* silencing did not affect the recruitment of total F4/80^+^ macrophages.

Remarkably, *Tie2* knockdown partially abrogated collateral vessel preconditioning and protection against ischaemic necrosis by *Phd*2^+/−^ macrophages, and limited ischaemia-induced arteriogenesis in WT mice (Fig 4A–D and Supporting Information Fig S4D). Consistently, *Tie2* knockdown reduced the fraction of MRC1^+^ M2-like macrophages in the adductors of *Phd2*^+/−^ amir(*Tie2*)-BMT mice at baseline as well as in WT amir(*Tie2*)-BMT and *Phd2*^+/−^ amir(*Tie2*)-BMT mice 7 days post-ligation (Fig 4E), without affecting the overall recruitment of F4/80^+^ macrophages (Fig 4F). These data indicate that increased levels of TIE2 are necessary to precondition *Phd*2^+/−^ macrophages at baseline and to skew WT macrophages towards a proarteriogenic phenotype following femoral artery occlusion.

### TIE2-expressing macrophages (TEMs) are required to fuel arteriogenesis in ischaemia

To assess if TEMs are required for the maturation of collateral arteries and thus preadaptation to ischaemia in *Phd2*^+/−^ mice, we used a suicide gene strategy based on the *Herpes simplex* virus thymidine kinase (tk)-ganciclovir (GCV) system described before (Capobianco et al, 2011; De Palma et al, 2003, 2005; Welford et al, 2011). We co-transduced WT or *Phd2*^+/−^ HSPCs with a LV expressing the tk cDNA under the control of the *Tie2* promoter/enhancer (Tie2:tk), which enables TEM elimination *in vivo*, and a LV ubiquitously expressing GFP from the *PGK* promoter (PGK:GFP), which enables measuring BM cell engraftment in the transplanted mice by scoring GFP expression (Supporting Information Table 1). Tranduced cells were transplanted in irradiated WT mice; by this approach, BM-derived TEMs can be specifically eliminated by GCV administration in the transplanted mice. By using flow cytometry of GFP^+^ cells and qRT-PCR analysis of integrated vectors in blood cells, we found that *Phd2* haplodeficiency in BM-derived haematopoietic cells did not inhibit their engraftment upon transplantation in irradiated mice (GFP^+^ cells, % of leukocyte population: 92.6 ± 2.5% in WT Tie2:tk-BMT mice and 89.3 ± 5.5% in *Phd2*^+/−^ Tie2:tk-BMT mice; *N* = 6; *p* > 0.05).

**Figure 5:**
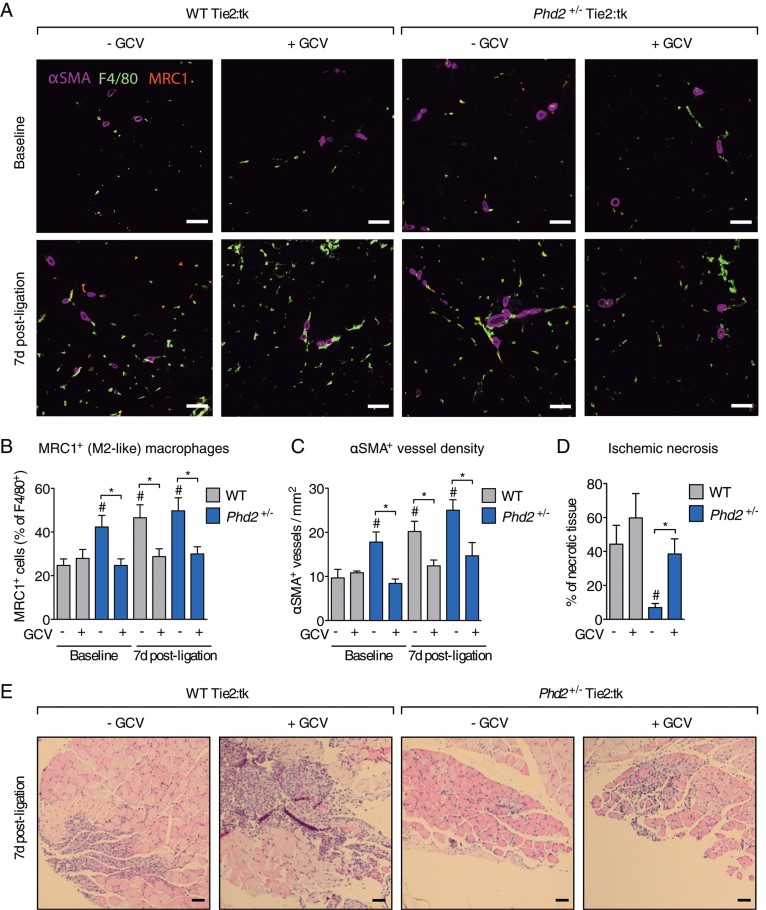
TEMs are required for ischaemic protection TEMs are specifically eliminated by administration of ganciclovir (GCV) in WT mice transplanted with HSPCs (from either WT or *Phd2*^+/−^ mice) transduced by a Tie2:tk LV. Analyses are performed at baseline and 7 days after femoral artery ligation (*N* = 5–8). *p < 0.05; ^#^*p* < 0.05 towards WT control baseline in B,C, towards WT control in D. Immunofluorescence staining for F4/80, MRC1, and αSMA on adductor sections (scale bars, 50 µm).Quantification of MRC1^+^ macrophages in adductors. The accumulation of M2-like proarteriogenic macrophages in *Phd2*^+/−^ mice at baseline or WT mice after ligation is abolished by TEM depletion.Quantification of αSMA^+^ vessels by immunofluorescence staining of adductors.Quantification of ischaemic necrosis in crural muscles (*N* = 4–5).H&E staining of crural muscles (scale bars, 100 µm).

**Figure 6:**
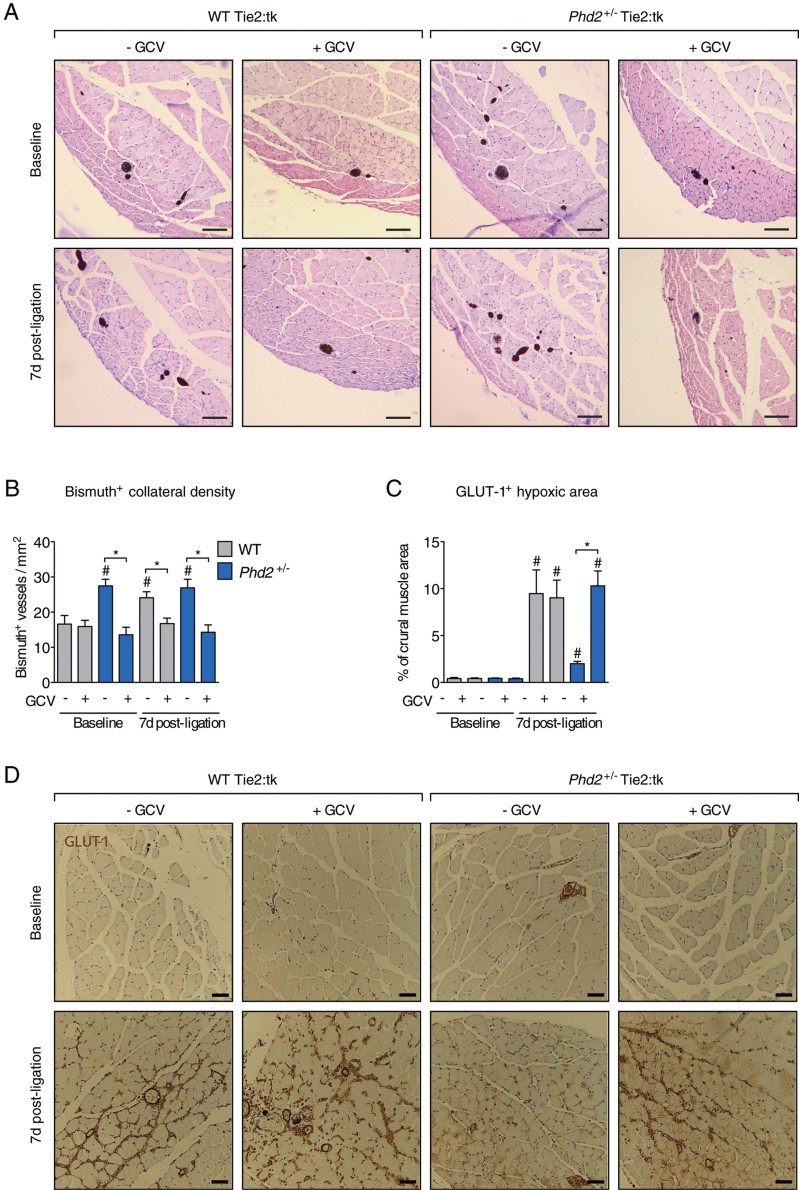
TEMs are functionally relevant for collateral maturation and perfusion *p < 0.05; ^#^*p* < 0.05 towards WT control in B, towards baseline in C. **A,B.** Quantification of perfused collateral vessels by H&E staining (A) or gelatin-bismuth based angiography (B) of adductors (*N* = 5–8; scale bars, 100 µm).**C.** Quantification of hypoxic tissue in crural muscles (*N* = 5–8).**D.** Immunostaining for GLUT-1 in crural muscles (scale bars, 50 µm).

We then studied the effects of TEM depletion in *Phd2* haplodeficient or -proficient BM chimeras. At baseline, F4/80^+^MRC1^+^ macrophages were reduced by 42% in GCV-treated *Phd2*^+/−^ Tie2:tk-BMT mice but not in GCV-treated WT Tie2:tk-BMT mice (Fig 5A and B). Seven days after femoral artery ligation, F4/80^+^MRC1^+^ macrophages were augmented by 1.9 times in vehicle-treated WT Tie2:tk-BMT mice, but GCV treatment almost completely prevented this increase (Fig 5A and B). These data are consistent with the expression of TIE2 in F4/80^+^ macrophages, and confirm that tissue-resident TEMs are scarce at baseline in WT mice but are increased in *Phd2*^+/−^ (preconditioned) mice. In ischaemia, TEMs were increased in WT but not *Phd2*^+/−^ mice, possibly because of preconditioning at baseline (Fig 5A and B). Remarkably, the arterial preconditioning and protection against ischaemic necrosis were completely abolished in GCV-treated *Phd2*^+/−^ Tie2:tk-BMT mice (Fig 5C–E and Supporting Information Fig S5A). In WT mice, TEM depletion by GCV administration prevented ischaemia-induced arteriogenesis measured 7 days post-ligation (Fig 5C). Consistent with the increased numbers of αSMA^+^ vessels, gelatin-bismuth angiographies showed the highest perfusion in the adductors of vehicle-treated *Phd2*^+/−^ Tie2:tk-BMT mice at baseline and of WT Tie2:tk-BMT mice 7 days post-ligation, effects that were greatly reduced by GCV-induced TEM depletion (Fig 6A and B). The reduction of collateral vessel formation consequent to TEM depletion aggravated lower limb ischaemia, as shown by increased staining for the hypoxia-induced glycoprotein GLUT-1 on crural muscle sections (Fig 6C and D). Despite hypoxia, the microvessel density in the ischaemic crural muscle was comparable to that at baseline at this time point of analysis, regardless of the experimental condition tested (Supporting Information Fig S5B and C). These data confirm that angiogenesis is delayed compared to arteriogenesis following femoral artery ligation (Luttun et al, 2002; Takeda et al, 2011). Likewise, vessel density in the adductors was comparable in all conditions (Supporting Information Fig S5B and C). Taken together, these results indicate that TEMs are required to support arteriogenesis following ischaemia.

## DISCUSSION

This study identifies a basic biological role for PHD2 in the control of a specific proarteriogenic macrophage phenotype that impinges on TIE2 signalling. Overall, the mechanisms described in this study may represent an indirect modality by which PHD2 modulates oxygen delivery through the regulation of vessel morphogenesis.

The proarteriogenic tissue macrophages identified here are highly reminiscent of the M2-like, proangiogenic TEMs found in tumours, developing organs and regenerating tissues (Capobianco et al, 2011; De Palma et al, 2005; Fantin et al, 2010; Mazzieri et al, 2011; Pucci et al, 2009). Despite the well-characterized proangiogenic functions of TEMs, no studies have yet been undertaken to rigorously assess the functional relevance of the ANG receptor TIE2 in macrophages in the context of ischaemia. We have previously shown that, similar to tumour-associated TEMs (Pucci et al, 2009), *Phd2*^+/−^ macrophages do not upregulate either *Vegf* or inflammatory genes, but instead express increased levels of *Tie2*, *Nrp1*, *Cxcr4*, *Pdgfb* and *Sdf1*, which together may enhance their proarteriogenic capacity (Takeda et al, 2011). These macrophages express high levels of *Ccr2* (Takeda et al, 2011), the CCL2 receptor, which has been shown to be one of the main signalling pathways for macrophage recruitment to the pericollateral area upon occlusion of a main arterial route (Fujii et al, 2006; Heil & Schaper, 2004; Ito et al, 1997b; Nickerson et al, 2009). While under non-pathological conditions *Tie2* expression is low in circulating monocytes; its expression is upregulated in perivascular, tumour-associated (De Palma et al, 2008; Pucci et al, 2009) or ischaemia-associated (this study and Patel et al, 2013) macrophages. Recent data have shown that ECs provide signals that can induce the differentiation of M2-like macrophages characterized by enhanced *Tie2* expression (He et al, 2012). After femoral artery occlusion, the bulk of blood flow is redirected into collateral conduits, generating shear stress that causes the release of various cytokines from vascular ECs and associated stromal cells. Stimulation of infiltrating WT macrophages by vessel-derived cytokines (*e.g.* ANG1) may induce the repression of *Phd2* transcription (to levels similar as in *Phd2*^+/−^ cells) and upregulation of *Tie2* in the macrophages. These data also imply that the abundance of PHD2, rather than oxygen availability, becomes the limiting factor for its activity in macrophages; of note, collateral formation is recognized as a hypoxia-independent process (Gray et al, 2007; Ito et al, 1997a). It remains an open question whether and how this mechanism is involved in the revascularization of the ischaemic (hypoxic) tissue, downstream the occlusion point.

**Figure 7:**
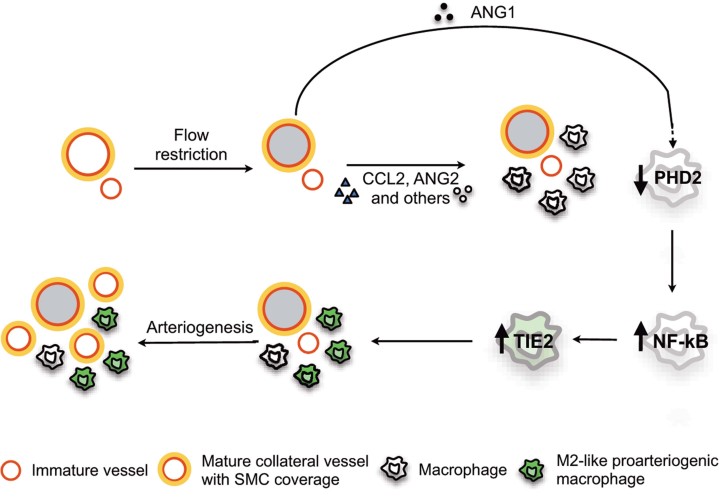
Schematic illustration of the key findings Flow restriction upon occlusion of a main arterial route results in downstream ischaemia and increases the shear forces on the vessel wall of upstream, preformed collateral vessels, inducing the release of different cytokines and chemokines (black triangles). Chemoattractant cues such as CCL2 (MCP1) and/or vessel permeability molecules, including ANG2 (white circles), promote − directly or indirectly − macrophage recruitment to the immature collaterals. Subsequently, ANG1 (black circles) induces transcriptional downregulation of *Phd2,* activating the NF-κB pathway. In turn, NF-κB upregulates *Tie2* in a feed-forward loop where TIE2 receptor is functionally required to sustain the *in situ* programming of macrophages towards a proarteriogenic, M2-like phenotype. TEMs are then required to promote the maturation of collateral vessels (arteriogenesis).

Previous studies have implicated ANG1 and ANG2 in the arteriogenenic process during ischaemia, although with opposing functions, possibly because the two cytokines may have different biological functions depending on the cellular context and/or experimental conditions (Lekas et al, 2012; Reiss et al, 2007; Shyu et al, 1998; Tressel et al, 2008). Recent data also suggest that recovery of vascular perfusion upon hind limb ischaemia is markedly impaired in mice deficient for the ANG receptor TIE2, and that a VEGF–ANG1 chimeric protein (but not a VEGF-ANG2 chimera) improves tissue perfusion after ischaemia (Anisimov et al, 2013; Lekas et al, 2012). Nevertheless, the direct role of the ANG–TIE2 axis in macrophages has not yet been studied in the context of ischaemia. Here, we have demonstrated that ANG1–TIE2 signalling favours the transcriptional repression of *Phd2* in tissue macrophages recruited after arterial occlusion, hence promoting their *in situ* skewing towards a proarteriogenic phenotype. Mechanistically, ANG1-mediated repression of *Phd2* in macrophages activates the canonical NF-κB pathway in normoxia, which induces *Tie2* upregulation; in turn, TIE2 enhances the proarteriogenic functions of macrophages (Fig 7). According to our results, *Phd2* haplodeficiency constitutively upregulates *Tie2* in macrophages, thus triggering the accumulation of proarteriogenic macrophages and enhancing collateral maturation at baseline. In line with previous literature, the peak of ANG2 occurring at 12 h after femoral artery occlusion may initiate vessel remodelling, loosen EC junctions, and indirectly induce monocyte/macrophage recruitment to the pericollateral space, possibly in combination with other chemoattractants, for example CCL2 (Lekas et al, 2012; Roviezzo et al, 2005). Then, constant high levels of ANG1 following femoral artery occlusion would sustain the *in situ* programming of macrophages towards an M2-like, proarteriogenic phenotype (Fig 7). Noteworthy, even when we overexpressed ANG2 to similar levels as ANG1, only the latter induced the MRC1^+^, M2-like phenotype in macrophages. Future studies should be aimed to identify the molecular pathway(s) contributing to ANG1-mediated *Phd2* inactivation.

Our finding that ANG1 initiates a genetic program in macrophages mirrors previous results showing that ANG2 regulates gene expression in TEMs and augments their proangiogenic functions in cancer and arthritis (Coffelt et al, 2010; Krausz et al, 2012). A recent study also supports a model wherein the ANG2-TIE2 axis mediates cell-to-cell interactions between TEMs and ECs during tumour angiogenesis (Mazzieri et al, 2011). Based on the above and our data, it is tempting to speculate that ANG1–TIE2 signalling in macrophages is instrumental to promote vessel maturation (this study), whereas ANG2-TIE2 signalling in macrophages is required to promote angiogenesis (Coffelt et al, 2010; Krausz et al, 2012; Mazzieri et al, 2011). Such dichotomic functions of TIE2 signalling in macrophages may parallel the better characterized functions of TIE2 signalling in ECs, wherein ANG1 induces cell–cell contacts and vessel stabilization, while ANG2 induces EC activation and angiogenesis (Augustin et al, 2009; Daly et al, 2013). The net outcome of TIE2 signalling may depend on the bioavailability of ANG1 and ANG2, whose expression are spatially and dynamically regulated during arteriogenic/angiogenic processes (Gerald et al, 2013; Saharinen & Alitalo, 2011; Saharinen et al, 2011).

Finally, our findings may have implications for cell-based therapies and suggest that TEMs might be transfused to promote collateral vessel growth (this study) or neovascularization (Patel et al, 2013) in patients with critical limb ischaemia or in patients at risk of ischaemic damage, for instance patients with diabetes or hypercholesteraemia.

## MATERIALS AND METHODS

For more detailled Materials and Methods see the Supporting Information.

### Animals

WT and *Phd2*^+/−^ Balb/C mice were obtained from our mouse facility. *Ikkb* conditional knockout mice were obtained from M. Karin (Chen et al, 2003) and intercrossed in our mouse facility with Lys:Cre floxed *Phd2* mice, as previously described (Takeda et al, 2011). Housing and all experimental animal procedures were performed in accordance with Belgian law on animal care and were approved by the Institutional Animal Care and Research Advisory Committee of the K. U. Leuven (P036/2009). In all experiments, littermates were used as controls.

### Mouse model of hindlimb ischaemia

To induce hind limb ischaemia, unilateral or bilateral ligations of the femoral artery and vein and the cutaneous vessels branching from the caudal femoral artery side branch were performed without damaging the nervus femoralis under anaesthesia with ketamine (1 × 75 mg/kg i.p.) and xylazine (1 × 5 mg/kg i.p.) (Luttun et al, 2002). By using this procedure, collateral flow to adductor muscles is directed via arterioles branching from the femoral artery, therefore preserving 50–60% of original perfusion. For analgesia, mice received titrated doses of buprenorphine (5 µg/kg i.p.).

### BMT and analysis of haematopoietic reconstitution

Balb/c WT recipient mice were irradiated with 7.5 Gy and transplanted with either total BM cells or BM-derived HSPCs from WT or *Phd2*^*+/−*^ donor mice. In some experiments, HSPCs were transduced with LVs to achieve silencing of *Tie2* in mature haematopoietic cells (Mazzieri et al, 2011), or to deplete TEMs using GCV (De Palma et al, 2005) (see Supporting Information Methods). The repopulating capacity of transduced HSPCs was analysed by FACS or by vector copy number (VCN) analysis.

### Gelatin-bismuth-based angiography

Gelatin-bismuth-based angiography was performed as described before (Takeda et al, 2011). After perfusion–fixation, the muscle tissue between the two superficial collateral arterioles (adductor) was post-fixed in 2% paraformaldehyde, paraffin-embedded and morphometrically analysed.

### Administration of sTIE2

BM-transplanted mice were injected both systemically (5 × 10^11^ vp in the tail vein) and locally (5 × 10^9^ vp directly in two sites of the adductor) with an AAV9 encoding the mouse extracellular domain of TIE2 fused to a flag tag (AAV9:sTIE2) with AAV9:Albumin as control. sTIE2 expression was analysed by Quantikine Mouse TIE2 ELISA kit (R&D).

### Overexpression of ANG1 and ANG2

WT mice were injected into the adductor muscle with an AAV9 (5 × 10^9^ vp) encoding ANG1 or ANG2, as previously described (Anisimov et al, 2013). Expression of ANG1 and ANG2 was assessed by qPCR on RNA extracted from adductors.

### Macrophage preparation

Macrophages were obtained from the peritoneal cavity or BM and silencing of the canonical subunits p65 (*Rela*) and p50 (*Nfkb1*) was achieved by electroporation with specific siRNAs, as previously described (Takeda et al, 2011). *In vitro* stimulation with recombinant ANG1 or ANG2 (Peprotech) was performed at a concentration of 250 ng/ml for 18 h.

### Histology and immunostaining

Tissue staining for necrosis in crural muscle, αSMA, F4/80, CD31 and MRC1, and their quantification, have been described before (Takeda et al, 2011). Hypoxic cells were analysed 2 h after injection of 60 mg/kg pimonidazole i.p., followed by staining with Hypoxyprobe Kit (Chemicon International) according to the manufacturer's instructions. Tissue oxygenation status was indirectly assessed by staining for GLUT-1 (Millipore) following the manufacturer's instructions. Apoptotic cells within and surrounding necrotic and hypoxic domains (Skuli et al, 2009) were identified by the TUNEL method using the ApopTag Kit (Millipore) following the manufacturer's instructions.

### Quantitative RT-PCR

Quantitative RT-PCR was performed using commercially available or home-made primers and probes listed in Supporting Information Methods.

### FACS analysis and macrophages cell sorting

Whole blood was stained for the pan-haematopoietic marker CD45, and OFP signal directly measured in the PE channel. F4/80^+^ macrophages were sorted from dissociated adductors, as previously described (Takeda et al, 2011).

### Statistics

The data were represented as mean ± SEM of the indicated number of measurements. Statistical significance was calculated by two-tailed unpaired *t*-test for two data sets and ANOVA followed by Bonferroni post hoc test for multiple data sets using Prism (GraphPad, Inc.), with *p* < 0.05 considered statistically significant.

**The paper explained**

PROBLEM:

Despite continuous progress in studying the physiological and pathological mechanisms underlying ischaemic diseases, none of the promising interventions in preclinical models have been translated into a convincing clinical application. We therefore sought to advance our previous studies on how to modulate macrophages in order to promote arteriogenesis, a process relying on the remodelling of preexisting vascular shunts that allows more rapid revascularization than hypoxia-driven angiogenesis.

RESULTS:

In this study, we present novel insight into oxygen-independent downregulation of the oxygen-sensitive enzyme, PHD2, controlled by the release of ANG1 following vascular occlusion. We show that the proarteriogenic macrophage phenotype fostered by PHD2 downregulation relies on ANG1–TIE2 signalling in macrophages. We further demonstrate the importance of the TIE2 receptor for the proarteriogenic functions of macrophages in the context of ischaemia, thus underscoring an indispensable role of TIE2-expressing macrophages in governing arteriogenesis and restoring oxygenation to downstream tissues.

IMPACT:

In light of the need for better therapies for ischaemic diseases, the molecular mechanisms regulating the proarteriogenic functions of macrophages may be harnessed to develop effective cell-based therapeutic approaches for the treatment of limb ischaemia. Our findings indeed suggest that cell-based therapies using PHD2 hypomorphic and/or TIE2-expressing macrophages might promote collateral vascularization in patients at risk of ischaemic damage, for instance patients with diabetes or hypercholesteraemia.

## Supplementary material

As a service to our authors and readers, this journal provides supporting information supplied by the authors. Such materials are peer reviewed and may be re-organized for online delivery, but are not copy-edited or typeset. Technical support issues arising from supporting information (other than missing files) should be addressed to the authors.
