# Supplementary material for: PHD2 regulates arteriogenic macrophages through TIE2 signalling
Source: EMBO Mol Med. 2013 Apr 25;5(6):843–57. doi: 10.1002/emmm.201302695 (PMC3779447; doi:10.1002/emmm.201302695)
Supplement: Supplementary file 1 [file emmm0005-0843-SD1.pdf]

EMBO Molecular Medicine

PHD2 regulates arteriogenic macrophages through TIE2 signaling

Alexander Hamm, Lorenzo Veschini, Yukiji Takeda, Sandra Costa, Estelle Delamarre, Mario Leonardo Squadrito, Anne-Theres Henze, Mathias Wenes, Jens Serneels, Ferdinando Pucci, Carmen Roncal, Andrey Anisimov, Kari Alitalo, Michele De Palma and Massimiliano Mazzone

Corresponding author: Massimiliano Mazzone, VIB Vesalius Research Center

Review timeline:

Submission date:	28 February 2013
Accepted:	12 March 2013

Transaction Report:

Please note that the manuscript was previously reviewed at another journal and the reports were taken into account in the decision making process at EMBO Molecular Medicine. Since the original reviews are not subject to EMBO's transparent review process policy, the reports and author response cannot be published.
