# Supplementary material for: PHD2 regulates arteriogenic macrophages through TIE2 signalling
Source: EMBO Mol Med. 2013 Apr 25;5(6):843–57. doi: 10.1002/emmm.201302695 (PMC3779447; doi:10.1002/emmm.201302695)
Supplement: Supplementary file 2 [file emmm0005-0843-SD2.pdf]

SUPPORTING INFORMATION

PHD2 regulates arteriogenic macrophages through TIE2 signaling

Alexander Hamm, Lorenzo Veschini, Yukiji Takeda, Sandra Costa, Estelle Delamarre, Mario Leonardo Squadrito, Anne-Theres Henze, Mathias Wenes, Jens Serneels, Ferdinando Pucci, Carmen Roncal, Andrey Anisimov, Kari Alitalo, Michele De Palma & Massimiliano Mazzone

TABLE OF CONTENTS

Methods	Page 3
Table 1	Page 8
Figures	Page 9
References	Page 14

SUPPORTING INFORMATION METHODS:

Bone marrow (BM) transplantation and hematological analysis: Balb/C WT recipient mice were irradiated with 7.5 Gy. Subsequently, 5 x 10^6 bone marrow cells from WT or *Phd2*$^{+/-}$ donor mice were injected intravenously via the tail vein. Femoral artery ligation was performed 6 weeks after bone marrow reconstitution. To assess reconstitution, red and white blood cell count was determined using a hemocytometer on peripheral blood collected in heparin with capillary pipettes by retro-orbital bleeding.

Hematopoietic stem/progenitor cell (HSPC) isolation, transduction and transplantation: Six to 12-week–old Balb/C mice were sacrificed and BM was harvested. Lineage negative cells (lin$^-$ cells) enriched in HSPCs were isolated from BM cells using a cell purification kit (StemCell Technologies) and transduced by concentrated LVs. Briefly, 10^6 cells/ml were pre-stimulated for 4-6 hours in serum-free StemSpan medium (StemCell Technologies) containing a cocktail of IL-3 (20 ng/ml), SCF (100 ng/ml), TPO (100 ng/ml) and FLT-3L (100 ng/ml) (all from Peprotech), and transduced with a LV dose equivalent to 10^8 LV Transducing Units/ml, for 12 hours in the same medium.

***Tie2* silencing in mature hematopoietic cells:** To silence *Tie2* expression specifically in mature hematopoietic cells, HSPC isolated as described above were transduced with LVs carrying an artificial microRNA either against *Tie2* (amiR(*Tie2*)) or a control microRNA targeting *Luciferase* (amiR(*Luc*)), as described above. Transduced cells were then superinfected with a second LV carrying the reverse tetracycline transactivator (rtTA-m2-miR-126T) (Mazzieri et al, 2011). Sixteen hours after transduction, 10^6 cells were infused into the tail vein of lethally irradiated, 6-week–old, female Balb/C mice (radiation dose: 7.5 Gy). Twenty-five days after irradiation, doxycyline (Sigma) or vehicle was administered three times a week at a dose of 25 mg per kg of body weight. Ligation was performed 6 weeks after irradiation.

TEM depletion: To deplete TEMs, we infused 6-week-old, lethally irradiated female Balb/C mice with 10^6 HSPCs co-transduced with two LVs, Tie2:tk (to deplete TEMs) and PGK:GFP (to label all BM-derived cells in transplanted mice), using LV doses equivalent to 10^8 LV Transducing Units/ml for each vector, as described above (De Palma et al, 2005). Depletion of TEMs was achieved by oral gavage of ganciclovir (GCV, 50 mg/kg daily or vehicle as control) 4 weeks after transplantation for 10 days before and 6 days after femoral artery ligation.

Administration of sTIE2: The BM of WT or *Phd2*$^{+/-}$ mice was transplanted into lethally irradiated WT mice. After 5 weeks, mice were injected both systemically (5x10^{11} vp in the tail vein) and locally (5x10^{9} vp directly in two sites of the adductor) with an AAV9 encoding the mouse extracellular domain of TIE2 (AAV-sTIE2) (Holopainen et al, 2012). AAV9 encoding for serum albumin (AAV-Alb) was used as control. One week after injection of the viral vector, mice were subjected to femoral artery ligation. Blood and adductor samples were harvested at baseline and 72h post-ligation and used to analyze bone marrow reconstitution by FACS or sort F4/80^{+}GFP^{+} macrophages, respectively.

Overexpression of ANG1 or ANG2: WT mice were injected locally (5x10^{9} vp directly in two sites of the adductor) with an AAV9 encoding ANG1 or ANG2 (Anisimov et al, 2013). AAV9:Albumin was used as negative control. 14 days after injection, mice were sacrificed, and adductors were harvested for FACS sorting of adductor macrophages or for RNA extraction from whole muscle.

Vector copy number analysis: Transduced HSPCs were cultured and collected after 9 days, whereas blood from the transplanted mice was collected 4 weeks after HSPC transplantation to measure the number of integrated LV copies/cell genome (vector copy number, VCN) by qRT-PCR, as previously described (De Palma et al, 2005). Briefly, for vector copy number (VCN) analysis, we performed qRT-PCR using custom TaqMan assays specific for β -actin, HSV-tk or HIV-gag sequences (Applied Biosystems). Standard curves for HSV-tk (contained by Tie2:tk LV) or HIV-gag (contained by both Tie2:tk and PGK:GFP LVs) were obtained from genomic DNA samples containing known amounts of integrated LV. The VCN of genomic DNA standard curves was determined using custom TaqMan assays specific for LVs (Applied Biosystems). The SDS 2.2.1 software was used to extract raw data (CT) and to perform VCN analysis. To calculate VCN we used the following formula: VCN = VCN(standard curve) * ng of HIV-gag or HSV-tk per ng of β-actin. The VCN of PGK:GFP LV was obtained by subtracting the VCN of HSV-tk from the total HIV-gag VCN.

ELISA quantification of sTIE2: sTIE2 was quantified in plasma using the Quantikine mouse TIE2 ELISA kit (R&D systems) according to the manufacturer's instructions. Plasma was prepared from blood samples that were collected at the time of sacrifice by retro-orbital bleeding.

Histology, immunostaining and morphometry: Adductor and crural muscles were dissected, fixed in 2% paraformaldehyde, dehydrated, embedded in paraffin, and sectioned at 7 μm thickness. Necrotic areas in the crural muscle were identified by staining the sections with haematoxylin and

eosin (H&E). Necrotic cells display a more glassy homogeneous appearance in the cytoplasm with increased eosinophilia, whereas the nuclear changes are reflected by karyolysis, pyknosis and karyorrhexis. Necrotic area was defined as the percentage of area that includes these necrotic myocytes, inflammatory cells and interstitial cells, compared to the total soleus area. After deparaffinization and rehydration, sections were blocked and incubated overnight with primary antibodies: mouse anti-αSMA Cy3 conjugated, dilution 1/500 (Sigma); rat anti-F4/80, dilution1/100 (Serotec); goat anti-MRC1, dilution 1/200 (R&D); rat anti-CD31, dilution 1/200 (BD); rabbit anti-GLUT-1, dilution 1/200 (Millipore). Sections were then incubated with appropriate secondary antibody: Donkey anti goat Alexa 647 (Molecular Probes); Donkey anti rat biotin conjugated. Development was done with fluorescent dyes or 3,3'-sidminobenzidine (DAB, Sigma). Slides were mounted with Prolong Gold Antifade with DAPI for nuclear staining (Invitrogen). Where needed, the fluorescent signal was further amplified by means of a Tiramide based amplification kit (TSA, Applied Biosystem). Slides were analyzed under a fluorescent microscope (Olympus BX41), and density and areas were calculated using the Cell^F software.

Macrophage preparation: To harvest peritoneal macrophages (pMØ), the peritoneal cavity was washed with 5 ml of RPMI 10% FBS. The pooled cells were then seeded in RPMI 10% FBS in 24-well plates ($5x10^5$ cells/well). After 6 hours of incubation at 37°C in a moist atmosphere of 5% CO2 and 95% air, non-adhering cells on each plate were removed by rinsing with phosphate-buffered saline (PBS). Adherent macrophages were cultured in RPMI 10% FBS for 16 hours. When higher amounts of cells were needed, macrophages were derived from bone marrow precursors (bone marrow derived macrophages, BMDM) as described before (Meerpohl et al, 1976). Briefly, bone marrow cells ($2x10^6$ cells/ml) were cultured in a volume of 5 ml in a 10 cm Petri dish (non tissue culture treated, bacterial grade) for 7 days in DMEM supplemented with 20% FBS and 30% L929 conditioned medium as a source of M-CSF. The cells obtained in those cultures are uniformly macrophages.

Macrophage electroporation: Silencing of the canonical pathway subunits p65 (*Rela*) and p50 (*Nfkb1*) was achieved by electroporation with specific siRNAs. Briefly, $2.4x10^6$ BM-derived macrophages were resuspended in 320 ml of Opti-MEM and were electroporated (250V, 950 mF, ∞Ω) with 60 pmol of siRNA for either scramble, p65, p50, or combination of p50 and p65. For higher efficiency of silencing, two different siRNA sequences for each respective gene were designed (http://rnaidesigner.invitrogen.com).

The siRNA sequences used are the following:

5’-TGTCTGCACCTGTTCCAAATT-3’ and 5’-TGCTGATGGAGTACCCTGATT-3’ for *p65* (NM_009045.4);

5’-GAATACTTCATGTGACTAATT-3’ and 5’-CAAAGGTTATCGTTCAGTTTT-3’ for *p50* (NM_008689.2);

5’-CACCGCTTAACCCGTATTGCCTAT-3’ for the scramble control.

Macrophage Stimulation: Peritoneal macrophages were allowed to adhere for 16 hours and then stimulated with recombinant angiopoietin-1 or angiopoietin-2, respectively (both from Peprotech) at 250 ng/ml for 18 hours. RNA was then extracted for qPCR analysis.

Quantitative PCR analysis: In order to investigate gene expression, quantitative RT-PCR (qRT-PCR) was performed. For gene expression studies in pMØ, the cells were cultured in normoxic condition for 16 hours and the RNA was extracted. To analyze the gene profile of adductor muscle, tissues were collected at the time points indicated and RNA was extracted. For adductor macrophages, F4/80$^+$ cells were sorted by FACS (see below) into lysis buffer, and RNA was extracted. Quantitative RT-PCR was performed from reversely transcribed cDNA, using commercially available primers from Applied Biosystems (Foster City, CA) with the following sequence IDs: *Ang1* (Mm00456498_m1), *Ang2* (Mm00545822_m1), *Ccl2/Mcp1* (Mm00441242_m1), *Nfkb1/p50* (Mm00476361_m1), *Rela/p65* (Mm00501346_m1), *Tek/Tie2* (Mm00443243_m1) and home-made primers and probes for *Egln1/Phd2* with the following sequences: probe, ACG-AAA-GCC-ATG-GTT-GCT-TGT-TAC-CCA; forward, GCT-GGG-CAA-CTA-CAG-GAT-AAA-C; reverse, CAT-AGC-CTG-TTC-GTT-GCC-T. mRNA levels of *Tie2* after inhibition of NF-kB pathway were measured by qRT-PCR on pMØ exposed for 12 hours to 500 nM 6-amino-4-(4-phenoxyphenylethylamino)quinazoline.

FACS analysis and macrophage cell sorting: FACS analysis was performed on 200 μl of peripheral blood, harvested by eye bleeding. Blood samples were incubated for 20 minutes at 4°C with anti-CD45-PerCP (BD-pharmingen) upon red blood cell lysis. For cell sorting of adductor macrophages, the adductors were dissected, dissociated mechanically and subsequently digested by incubation in collagenase I solution for 45 minutes at 37°C, followed by dispase solution for 15 minutes at 37°C. The digested cell suspension was incubated for 15 minutes with Mouse BD Fc Block™ purified anti-mouse CD16/CD32 mAb (BD-pharmingen) and stained with rat

Alexa488-conjugated anti-F4/80 antibody (Serotec) for 20 minutes at 4°C. Adductor macrophages were sorted as F4/80$^+$ cells by gating against isotype IgG as negative control.

SUPPORTING INFORMATION TABLE 1:

Vector copy number in blood cells of WT Tie2:tk-BMT and *Phd2*$^{+/-}$ Tie2:tk-BMT mice.

	HSV-tk	HIV-gag	PGK:GFP
**WT Tie2:tk-BMT**	8.93 ± 0.40	15.79 ± 0.02	6.85 ± 0.43
***Phd2*$^{+/-}$ Tie2:tk-BMT**	10.53 ± 0.30	19.39 ± 0.01	8.85 ± 0.31

The data represent the number of integrated LV copies per cell genome (vector copy number, VCN ± SEM) of HSV-tk and HIV-gag in blood cells, collected 4 weeks after transplantation from WT Tie2:tk-BMT and *Phd2*$^{+/-}$ Tie2:tk-BMT mice. The VCN of PGK:GFP was obtained by subtracting the VCN of HSV-tk from the total HIV-gag VCN (N=6; P>0.05). See Supporting Information Methods for technical details.

SUPPORTING INFORMATION FIGURES:

Supporting Information Figure S1

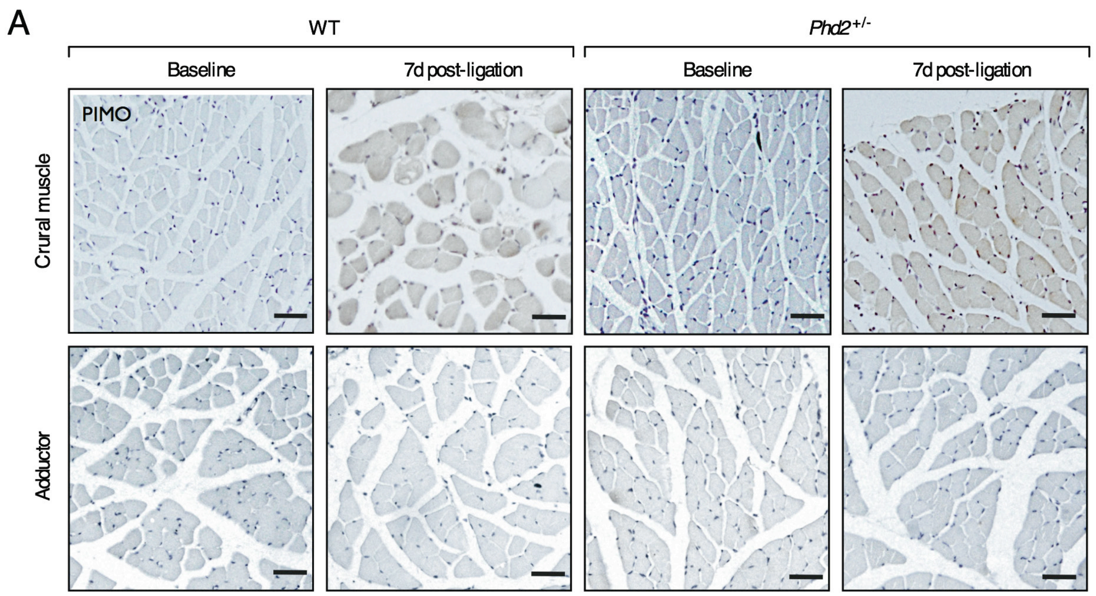

Supporting Information Figure S1: Femoral artery ligation induces detectable levels of hypoxia in the crural muscle but not in the adductor

A. Hypoxyprobe staining (brown) of crural muscles and adductors upon injection of pimonidazole in WT and *Phd2*$^{+/-}$ mice (N = 4-5; scale bars, 50 μm).

Supporting Information Figure S2

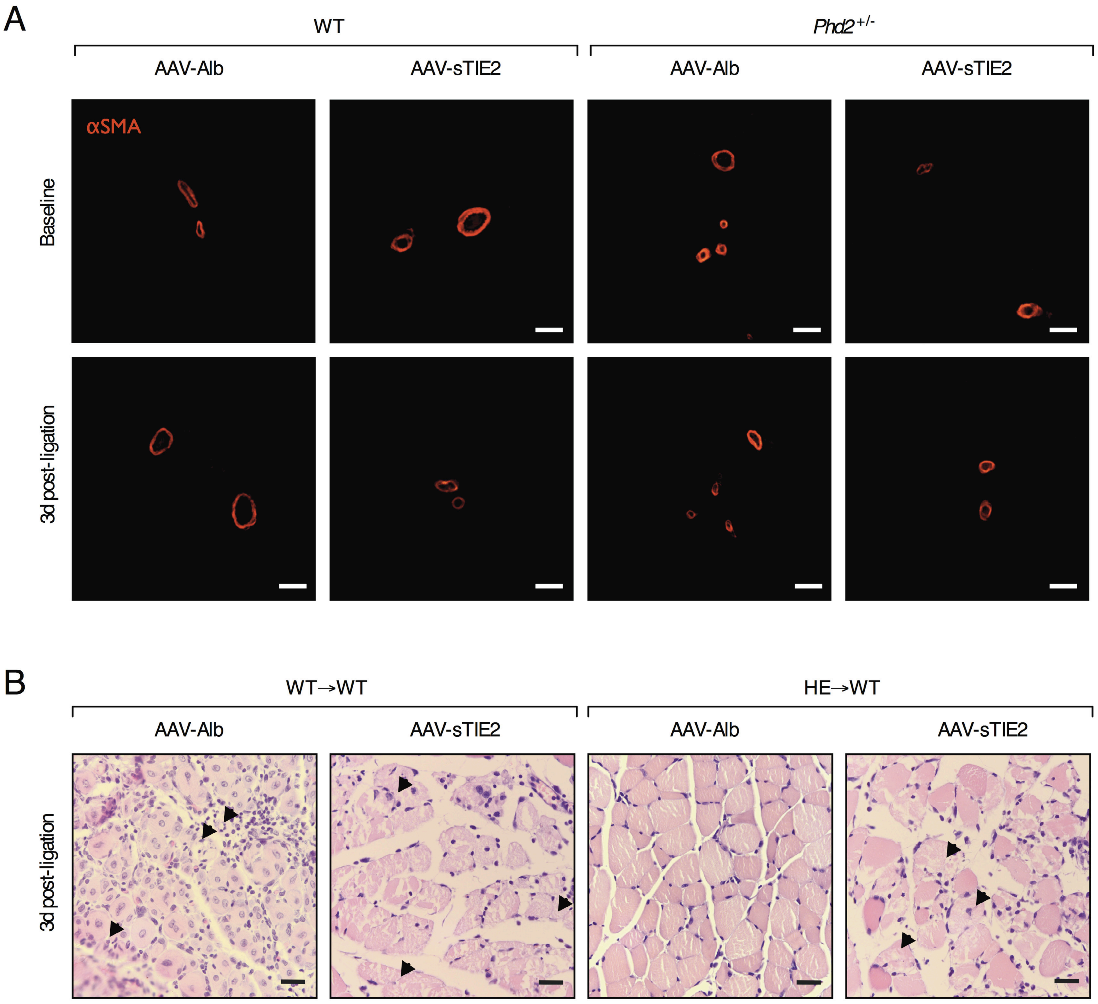

Supporting Information Figure S2: ANG blockade abrogates collateral vessel preconditioning and protection from ischemic necrosis

A. Immunofluorescence staining for αSMA. αSMA^+ vessels in adductor sections upon administration of an AAV encoding for a soluble ANG trap (AAV-sTIE2; indicated as [+]) or albumin as control (AAV-Alb; indicated as [-]) in WT mice transplanted with BM cells from WT (WT→WT) or *Phd2*$^{+/-}$ (HE→WT) mice, both at baseline and 3 days after ligation. (N=4-5; scale bars, 50 μm). See Figure 2A for quantification.

B. H&E staining of ischemic necrosis, representing a high-power magnification of Figure 2F (N=4-5; scale bars, 50 μm; arrowheads, necrotic cells).

Supporting Information Figure S3

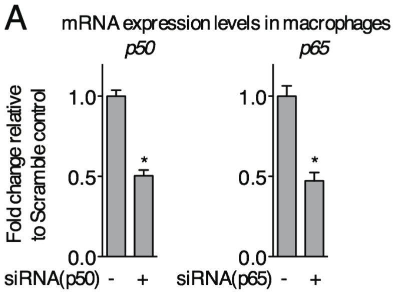

Supporting Information Figure S3: Silencing of p50 and p65 in peritoneal macrophages

A. Silencing efficiency by siRNA for the canonical subunits p65 (*Rela*) or p50 (*Nfkb1*) in peritoneal macrophages (N=4; *, P<0.05).

Supporting Information Figure S4

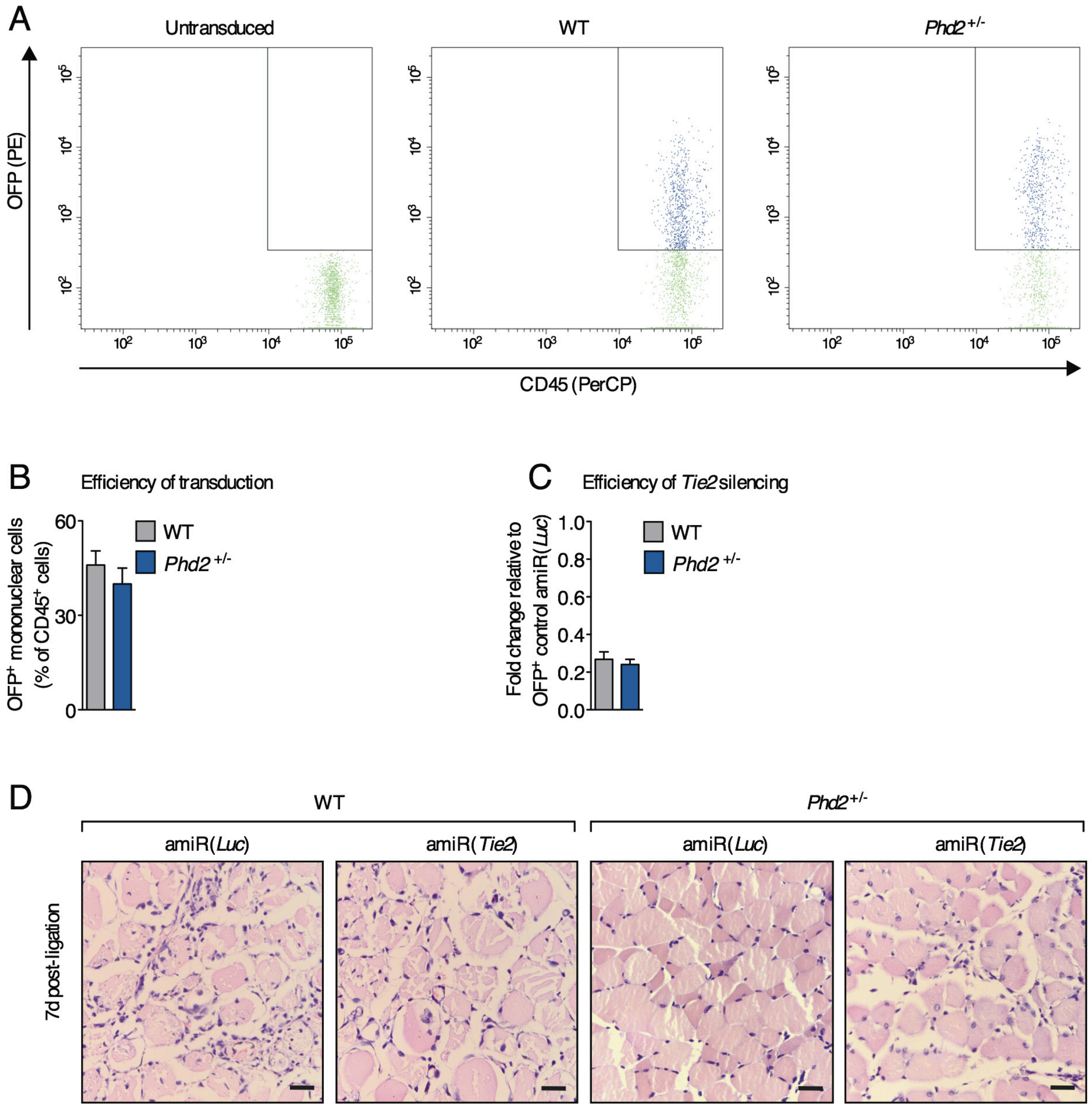

Supporting Information Figure S4: *Tie2* silencing in mature hematopoietic cells

WT mice were transplanted with HSPCs from WT or *Phd2*$^{+/-}$ mice, expressing an artificial miRNA against *Tie2* (amiR(*Tie2*)) or a control amiR (amiR(*Luc*)) (see methods section for details).

A. FACS staining for CD45 (PerCP) and OFP fluorescence on blood harvested from transplanted mice.

B. Quantification of hematopoietic reconstitution by OFP$^+$ cells expressing the amiR (N=18-20).

C. *Tie2* mRNA levels in OFP$^+$ adductor macrophages, expressed as fold change in macrophages expressing the amiR(*Tie2*) compared to the control amiR(*Luc*) (N=8-10).

D. H&E staining of ischemic necrosis, representing a high-power magnification of Figure 4D (N=4-5; scale bars, 50 μm).

Supporting Information Figure S5

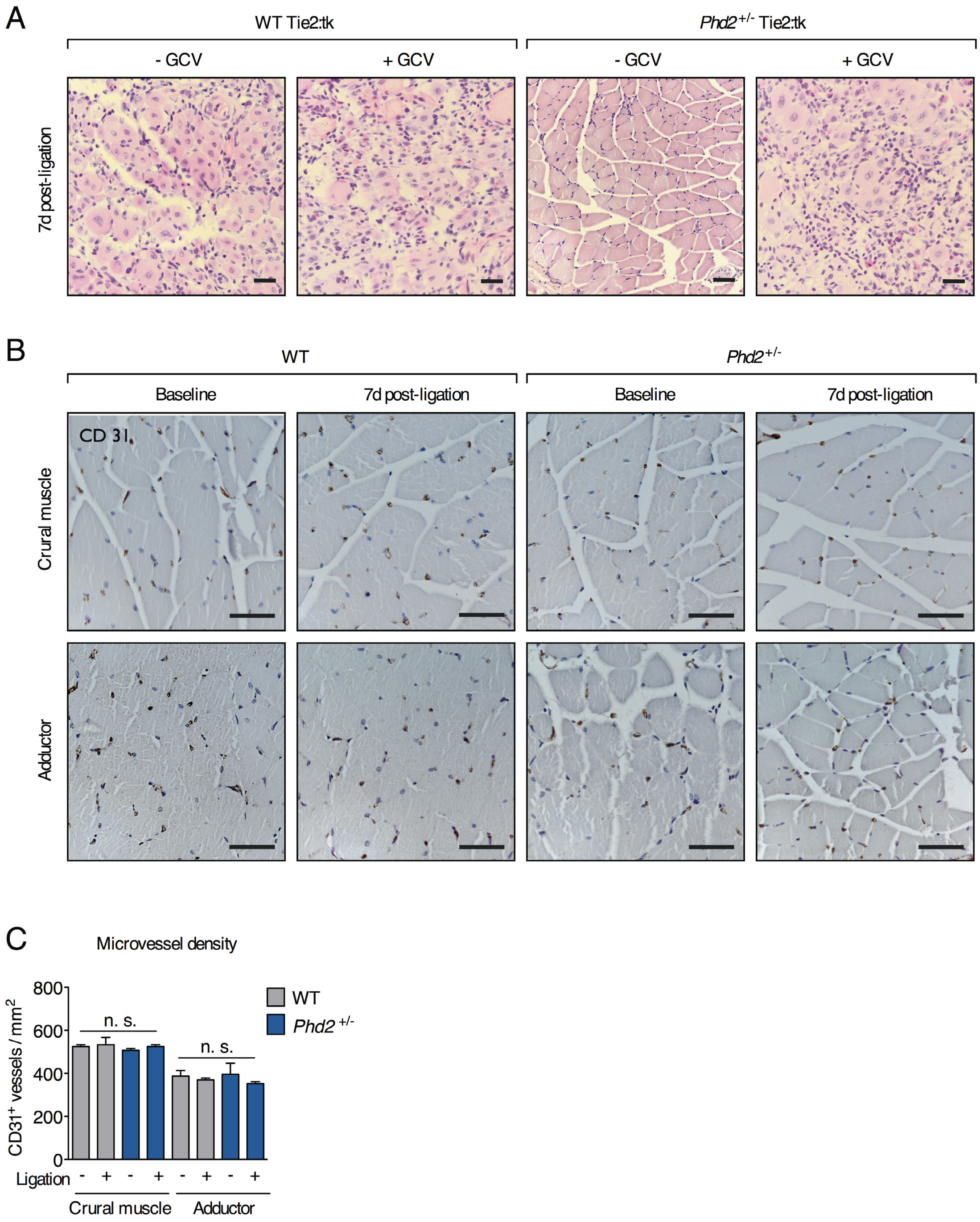

Supporting Information Figure S5: Microvessel density is unaffected early after ligation

A. H&E staining of ischemic necrosis after TEM elimination by ganciclovir (GCV) in WT mice transplanted with HSPCs from WT or *Phd2*$^{+/-}$ mice, expressing the Tie2:tk LV. Images represent high-power magnification of Figure 5E (N=4-5; scale bars, 50 μm).

B. Representative images and

C. quantification of microvessel density in WT and *Phd2*$^{+/-}$ mice at baseline and 7 days post-ligation, performed on sections of adductor and crural muscles stained for the endothelial cell specific marker CD31 (N=4-5; scale bars, 50 μm; n.s., not significant (P>0.05)).

SUPPORTING INFORMATION REFERENCES

Anisimov A, Tvorogov D, Alitalo A, Leppanen VM, An Y, Han EC, Orsenigo F, Gaal EI, Holopainen T, Koh YJ et al (2013) Vascular endothelial growth factor-angiopoietin chimera with improved properties for therapeutic angiogenesis. Circulation 127**:** 424-434

De Palma M, Montini E, Santoni de Sio FR, Benedicenti F, Gentile A, Medico E, Naldini L (2005) Promoter trapping reveals significant differences in integration site selection between MLV and HIV vectors in primary hematopoietic cells. Blood 105**:** 2307-2315

Holopainen T, Saharinen P, D'Amico G, Lampinen A, Eklund L, Sormunen R, Anisimov A, Zarkada G, Lohela M, Helotera H et al (2012) Effects of angiopoietin-2-blocking antibody on endothelial cell-cell junctions and lung metastasis. Journal of the National Cancer Institute 104**:** 461-475

Mazzieri R, Pucci F, Moi D, Zonari E, Ranghetti A, Berti A, Politi LS, Gentner B, Brown JL, Naldini L et al (2011) Targeting the ANG2/TIE2 axis inhibits tumor growth and metastasis by impairing angiogenesis and disabling rebounds of proangiogenic myeloid cells. Cancer Cell 19**:** 512-526

Meerpohl HG, Lohmann-Matthes ML, Fischer H (1976) Studies on the activation of mouse bone marrow-derived macrophages by the macrophage cytotoxicity factor (MCF). Eur J Immunol 6**:** 213-217
